# What, how and from whom do health care professionals learn during collaboration in palliative home care: a cross-sectional study in primary palliative care

**DOI:** 10.1186/s12913-014-0501-9

**Published:** 2014-11-07

**Authors:** Peter Pype, Wim Peersman, Johan Wens, Ann Stes, Bart Van den Eynden, Myriam Deveugele

**Affiliations:** Department of Family Medicine and Primary Health Care, Ghent University, UZ-6K3, De Pintelaan 185, Gent, 9000 Belgium; Department: Primary and Interdisciplinary Care Antwerp - PICA, Institution: University of Antwerp, Antwerp, 2610 Belgium; Institute for Education and Information Sciences, Institution: University of Antwerp, Antwerp, 2610 Belgium

**Keywords:** Workplace learning, Interdisciplinary communication, Physician-nurse relations, Primary health care, Palliative care

## Abstract

**Background:**

Palliative care often requires inter-professional collaboration, offering opportunities to learn from each other. General practitioners often collaborate with specialized palliative home care teams. This study seeks to identify what, how and from whom health care professionals learn during this collaboration.

**Methods:**

Cross-sectional survey in Belgium. All palliative home care teams were invited to participate. General practitioners (n = 267) and palliative care nurses (n = 73) filled in questionnaires.

**Results:**

General practitioners (GPs) and palliative care nurses learned on all palliative care aspects. Different learning activities were used. Participants learned from all others involved in patient care. The professionals’ discipline influences the content, the way of learning and who learns from whom. Multiple linear regression shows significant but limited association of gender with amount of learning by GPs (M < F; p = 0.042; Adj R^2^ = 0.07) and nurses (M > F; p = 0.019; Adj R^2^ = 0.01).

**Conclusions:**

This study is the first to reveal what, how and from whom learning occurs during collaboration in palliative care. Training professionals in sharing expertise during practice and in detecting and adequately responding to others’ learning needs, could optimize this way of learning.

**Electronic supplementary material:**

The online version of this article (doi:10.1186/s12913-014-0501-9) contains supplementary material, which is available to authorized users.

## Background

Palliative care is complex care. To address different needs of palliative patients and their families, interdisciplinary collaboration is advised [1]. When caring for terminally and chronically ill patients at home, collaborative practice results in higher satisfaction, fewer clinic visits, fewer symptoms and patients’ overall improved health [2]. Joining competencies of professionals from different disciplines in a well-organized home care team results in a more comprehensive and holistic approach [3]. Care coordination and interdisciplinary teamwork has been listed as one of the ten core competencies in palliative care [4,5]. In primary care, general practitioners (GPs) often collaborate with specialized palliative home care teams (PHCTs), resulting in high quality palliative care [6,7]. Besides improving patient care quality, working together offers learning opportunities where professionals learn with, from and about each other [8-11]. Knowledge and expertise is shared and professionals not only ‘learn from’ each other but also ‘teach’ each other in a reciprocal way: workplace learning (WPL). Many known definitions of WPL state the following aspects: mostly informal, embedded in daily practice, requires personal engagement, and knowledge is socially constructed [12-16]. Furthermore WPL is driven by actual learning needs, offers immediate possibility to put learning into practice, is a continuous and natural process which requires less or no planning, and (peer) mentors are readily available [9,17-19]. Therefore WPL might be a valuable complement to current education and training for health care professionals as it seems to address knowledge gaps and skills required for patient care directly. Throughout undergraduate and graduate training, practice experience has proven to be of value and is generally accepted and promoted. The importance of inter-professional WPL during professionals’ careers is less clear. The literature provides no benchmark on how much and in what way professionals learn during inter-professional collaboration. Therefore, before promoting this way of learning, we need to further explore it, as it is currently unknown to what extent (how much and in what way) WPL occurs in primary palliative care (care for palliative patients in primary health care, i.e., by GPs and the primary health care team). This study aims to Explore the Learning Impact of Collaboration in Inter-professional health care Teams (ELICIT-study). We draw on existing standards (e.g., the European Association for Palliative Care curriculum suggestions for content) and theoretical frameworks (e.g., a typology of workplace learning activities) to decide on the selected variables. This will further be explained in the Methods section. This study seeks to fill the literature gap by answering the following research questions (RQ):

### Primary questions

- RQ 1: What do GPs and PHCT nurses learn during collaborative practice?
- RQ 2: How do GPs and PHCT nurses learn during collaborative practice?
- RQ 3: From whom do GPs and PHCT nurses learn during collaborative practice?

### Secondary questions

- RQ 4: Is there an association between what, how and from whom GPs and PHCT nurses learn during collaborative practice?
- RQ 5: How much do GPs and PHCT nurses learn during collaborative practice?
- RQ 6: How much do personal characteristics influence the amount of learning during collaborative practice?

## Methods

### Design

A cross-sectional design was used.

### Settings, sample and procedure

In Belgium, GPs often collaborate with PHCTs. Specialized team nurses visit the palliative patient at home. A palliative care physician and a psychologist make up the rest of the team and support the nurses in their task during team meetings without making home visits themselves. The GPs’ main contact with PHCTs is through the nurses via telephone or through joint home visits. The Dutch speaking part of Belgium is covered by 15 PHCTs. All fifteen were asked to participate. All patients (taken care of by the PHCTs) who died during a three month period (May–July 2012) were included in the study but did not participate themselves. The attending GP and PHCT nurse were asked to fill in online questionnaires for each patient, immediately after the patient’s death. Every GP and PHCT nurse received a form with log-in code for the web survey. Each PHCT assigned this task of handing over the form to the participants to one person (might be a nurse or an administrator). A written informed consent was obtained by all participants. Ethics approval was obtained from the Ghent University Hospital (B670201213298).

### Questionnaires

- Demographics and personal characteristics of participating GPs and nurses (age, gender, profession, type of practice, years in practice, previous education in palliative care) were registered (RQ6).
- To answer the question whether and what participants learned, a list was presented with palliative care topics based on the postgraduate curriculum suggestions of the European Association for Palliative Care (EAPC) (physical items: 28 questions; psychosocial items: 29 questions; religious and cultural items: 7 questions; teamwork: 2 questions; care set-up: 7 questions) [20]. The list was edited by one author (mirroring the content of the curriculum suggestions) and consequently commented on and approved by a team of GPs, palliative care physicians and medical educators by email. The final version of the questionnaire has been discussed and approved by the authors (GPs, palliative care physicians and a psychologist). Participants were asked to answer ‘yes’ or ‘no’ to indicate if they had learned anything during the previous collaboration. These answers allowed us to count the items learned per participant and per collaboration period (i.e., starting from the day the GP asks for the PHCT’s support until the patient died) (RQ1 and 5). This means that the scores can range between 0 and 73 items learnt for each participant. See Additional file 1 for the list.
- On the “how” question, a list of eight possible learning activities was presented based on Eraut’s typology of workplace learning [16]. These activities are: Asking questions (e.g., ‘Can I combine morphine with scopolamine in a syringe driver?’); Getting information (e.g., ‘GP received a hard copy of the new guideline on pain treatment to inform him on how clinical reasoning can be done’); Locating resource people (e.g., ‘GP received the phone number of a palliative care specialist in answer to a complex question’); Listening and observing (e.g., ‘GP was present when the PHCT nurse had a difficult conversation with the patient. He learned a new way of addressing a patient’s fear’); Reflecting (e.g., ‘Deliberation between GP and palliative care nurse over drug regimen to minimize side effects’); Learning from mistakes (e.g., ‘GP made a mistake when calculating the equivalent dose between oral morphine and transdermal fentanyl. The patient was stuporous afterwards and the nurse explained the correct way of calculating’); Receiving feedback (e.g., ‘GP questioned the patient on his pain syndrome. Afterwards the PHCT nurse explained to him what other questions could have been asked’); and Use of mediating artefacts (e.g., ‘GP received a tool for pain measurement he was not used to working with’). A list of these activities was accompanied by clarifying examples. For each acquired topic, they were asked to denote the learning activity they used (RQ2). For each learning activity, a percentage was given of the total amount of learning activities used.
- On the “from whom” question, a list was presented with all health care providers present (primary care and hospital based) as well as the patient and his/her family. For each learned topic, they were asked to indicate from whom they had learned it (RQ3). For each of the persons involved, a percentage was given of the total amount of times persons have been addressed.
- In order to clarify which variables influence the total amount of learning, demographics of the participants and the Readiness for Inter-professional Learning Scale (RIPLS) were used. This is a 23-item scale with three factors: 1. Teamwork and Collaboration; 2. Patient Centeredness; 3. Sense of Professional Identity. The scale has been validated for use in primary care and examines the attitudes of health care professionals towards inter-professional learning [21-23]. Each item has to be scored on a 5-point Likert scale. A higher score is associated with higher readiness for inter-professional learning (scores can range between 23 and 135) (RQ6).

All questionnaires were pre-tested for feasibility and understanding using cognitive interviews [24]. Interviewees in two rounds were GPs (n = 8) and nurses (n = 8), not involved as participants in the study.

### Analysis

Analysis was done using SPSS v.20. Descriptive statistics (mean, standard deviation) were calculated for gender, age, previous palliative care education, type of practice, years in practice, RIPLS score, number of items learned, content of learning, learning activity and source of learning. The content of learning, learning activity and source of learning are calculated and reported as a percentage of total number of learned items, learning activities and sources of learning respectively (RQ1, 2 and 3). Cross-tabs are drawn to show the association between what, how and from whom GPs and PHCT nurses learn during collaborative practice (RQ4). Chi squared test was used to show associations between professional group and the content of learning, learning activities and sources of learning (RQ1, 2 and 3). Descriptive statistics (mean, median, standard deviation, minimum and maximum) were calculated for the total number of items learned (RQ5). An independent sample t-test was conducted to compare the total count of learned items between GPs and PHCT nurses, between males and females and between GPs with or without previous palliative care education (RQ6). A one-way ANOVA was used to test the effect of type of practice and age category on the number of items learned (RQ6). Simple linear regression was used to evaluate the effect of the RIPLS score on the number of items learned and years in practice on the number of items learned (RQ6). Multiple linear regression analysis was used to gauge the influence of participants’ gender, age, previous palliative care education, type of practice, years in practice and RIPLS score on the number of items learned (RQ6).

## Results

### Participants

Twelve out of 15 PHCTs agreed to participate. During the three-month registration period, 267 GPs (42.7%, no GPs with multiple questionnaires) and 73 PHCT nurses (100% of the 73 nurses working in the region at the time of the study; only the first questionnaire of each nurse was included) each completed one questionnaire. Characteristics of participants are shown in Table 1.

**Table 1:** **Characteristics of participants**

	**GPs (n = 267)**
**Gender**	
**Male**	185 (69.3%)
**Female**	78 (29.2%)
**Missing**	4 (1.5%)
**Age category**	
**< 31**	17 (6.4%)
**31–40**	31 (11.6%)
**41–50**	68 (25.4%)
**51–60**	91 (34.1%)
**> 60**	60 (22.5%)
**Type of practice**	
**Solo**	120 (44.9%)
**Duo**	60 (22.5%)
**Group**	83 (31.1%)
**Missing**	4 (1.5%)
**Pall care education**	
**Yes**	59 (22.1%)
**No**	203 (76.0%)
**Missing**	5 (1.9%)
	**PHCT nurses (n = 73)**
**Gender**	
**Male**	14 (19.2%)
**Female**	57 (78.1%)
**Missing**	2 (2.7%)
**Age category**	
**< 31**	9 (12.3%)
**31–40**	28 (38.4%)
**41–60**	32 (43.8%)
**> 60**	4 (5.5%)
**Years in practice**	
**Mean**	7.15
**Standard deviation**	5.1

### What do GPs and PHCT nurses learn during collaborative practice? (RQ1)

Both GPs and PHCT nurses learned most about psychosocial and physical issues. The percentages differed in a statistically significant way (p = 0.001). GPs learned about psychosocial (40.6%) and physical (35.5%) items in an almost equal number. Nurses mainly learned about psychosocial items (50.9%) and secondly about physical items (26.2%). Religious and spiritual items (13.2%/11.6%), teamwork items (7.0%/9.2%) and organizational items (3.6%/2.1%) were much less mentioned by GPs and nurses, respectively.

### How do GPs and PHCT nurses learn during collaborative practice? (RQ2)

Both groups of professionals listed the same two learning activities as the ones most used: ‘discussion and reflection’ and ‘listening and observing’. Percentages however differed significantly between professions (p <0.001). GPs stated they learn most by discussion and reflection (29.4%) and by listening and observing (28.2%). Learning from mistakes (3.0%) and using mediating artefacts (1.8%) were the least mentioned. Nurses predominantly learned by listening and observing (37.0%), followed by discussion and reflection (19%). Learning from mistakes (1.0%) and using mediating artefacts (1.5%) were the least mentioned.

### From whom do GPs and PHCT nurses learn during collaborative practice? (RQ3)

GPs as well as PHCT nurses indicated patients and their family members as the most frequent source of information. Percentages however differed in a statistically significant way (p <0.001). GPs mostly learned from patient and family (38.3%), from PHCT nurses (29.2%) and through self-study (10.5%). PHCT nurses learned from patient and family (47.6%), others (14.4%) and GPs (9.9%).

**Figure 1:**
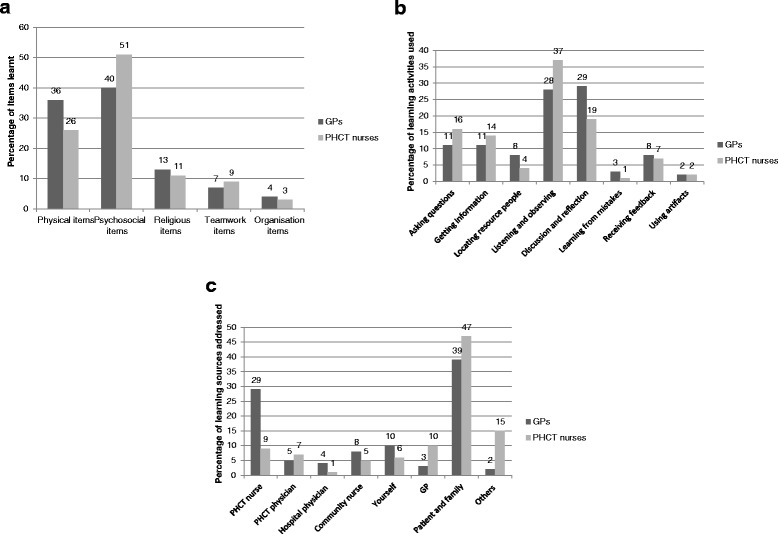
**Features of learning during collaborative practice. a**: Learning content by GPs and PHCT nurses (%). **b**: Which learning activities are used (%) by GPs and PHCT nurses. **c**: Who do GPs and PHCT nurses learn from.

An overview is shown in Figure 1.

### Is there an association between what, how and from whom GPs and PHCT nurses learn during collaborative practice? (RQ4)

Participants reported using different learning activities and addressing different sources of learning according to the topic in question. Patient-related topics and non-patient related topics (like ‘teamwork’ and ‘organization’) seem to differ in this. The row percentages of Table 2 shows the percentage of learning activities used and the percentage of learning sources addressed for each category of learning content.

**Table 2:** **Way of learning and source of learning according to learning topic for GPs and PHCT nurses (row percentages)**

**Way of learning/learning topic**	**Asking questions**	**Getting information**	**Locating resource people**	**Listening and observing**	**Discussion and reflection**	**Learning from mistakes**	**Receiving feedback**	**Use of mediating artifacts**
**General Practitioners**								
**Physical topics**	12.60%	16.30%	12.10%	13.80%	29.00%	3.80%	8.70%	3.70%
**Psychosocial topics**	6.70%	4.30%	3.30%	42.00%	32.00%	3.80%	7.80%	0.20%
**Religious – cultural topics**	20.10%	19.10%	5.30%	46.90%	2.40%	0.50%	5.30%	0.50%
**Teamwork topics**	7.60%	10.80%	12.10%	53.50%	0.00%	1.30%	12.70%	1.90%
**Organizational topics**	15.40%	35.20%	18.70%	20.90%	0.00%	0.00%	8.80%	1.10%
**PHCT nurses**								
**Physical topics**	17.20%	15.10%	6.50%	17.20%	30.10%	1.10%	9.70%	3.20%
**Psychosocial topics**	15.10%	11.20%	2.40%	48.60%	16.70%	1.20%	4.40%	0.40%
**Religious – cultural topics**	23.10%	23.10%	11.50%	23.10%	0.00%	0.00%	11.50%	7.70%
**Teamwork topics**	7.10%	7.10%	7.10%	28.60%	0.00%	0.00%	42.90%	7.10%
**Organizational topics**	0.00%	60.00%	0.00%	40.00%	0.00%	0.00%	0.00%	0.00%
**Source of learning/learning topic**	**PHCT nurse**	**PHCT physician**	**Hospital physician**	**Community nurse**	**Yourself**	**GP**	**Patient and family**	**Others**
**General Practitioners**								
**Physical topics**	47.80%	9.00%	6.90%	10.80%	7.30%	3.70%	13.10%	1.40%
**Psychosocial topics**	13.60%	1.60%	2.20%	6.50%	13.60%	2.60%	58.50%	1.40%
**Religious – cultural topics**	11.00%	7.90%	2.80%	1.00%	7.90%	6.60%	55.50%	7.20%
**Teamwork topics**	20.50%	4.90%	2.70%	35.20%	1.90%	19.70%	8.30%	6.80%
**Organizational topics**	16.00%	3.40%	5.90%	9.20%	10.90%	4.20%	24.40%	26.10%
**PHCT nurses**								
**Physical topics**	16.90%	22.50%	0.00%	4.50%	5.60%	9.00%	33.70%	7.90%
**Psychosocial topics**	8.00%	0.70%	0.70%	0.70%	7.30%	7.30%	58.50%	16.70%
**Religious – cultural topics**	12.90%	16.10%	0.00%	0.00%	3.20%	6.50%	41.90%	19.40%
**Teamwork topics**	3.40%	10.30%	0.00%	41.40%	0.00%	31.00%	6.90%	6.90%
**Organizational topics**	0.00%	14.30%	0.00%	14.30%	14.30%	0.00%	28.60%	28.60%

### How much do GPs and PHCT nurses learn during collaborative practice per palliative care experience (i.e., the collaboration in the care for one patient)? (RQ5)

General practitioners reported a mean total number of items learned of 5.1 (SD = 4.1; Median = 4.0; range = 0–16)) and PHCT nurses of 4.6 (SD = 3.8; Median = 4.0; range = 0–16)). There was no significant difference between the means of the two professional groups (p = 0.302).

### Which variables influence the amount of learning during collaborative practice? (RQ6)

#### Bivariate analysis

Female GPs indicated learning more often (p = 0.01) during collaboration (M = 6.15; SD = 4.31) than male GPs (M = 4.73; SD = 3.99). Previous palliative care education did not affect the results.

For PHCT nurses there was no significant difference in gender in the count of items.

There was no significant difference in count for age category (for GPs and PHCT nurses) and type of practice (for GPs).

There was a significant effect on RIPLS score on total count of items learned for GPs; p = 0.024 (higher score on RIPLS associated with more items learned) but not for PHCT nurses.

For PHCT nurses there was a significant difference in the number of items learned for years in practice p = 0.041 (more years in practice associated with less items learned).

These results are shown in Table 3.

**Table 3:** **Factors associated with total count of items learned – bivariate analysis**

	**N**	**Mean (SD)**	**P**
**Profession**			
**GP**	**267**	**5.1 (4.1)**	**NS**
**PHCT nurse**	**73**	**4.6 (3.8)**	
	**GPs (n = 267)**	**Mean (SD)**	**P**
**Gender**			
**Male**	185 (69.3%)	4.7 (4.0)	0.01
**Female**	78 (29.2%)	6.1 (4.3)	
**Missing**	4 (1.5%)		
**Age category**			
**< 31**	17 (6.4%)	7.1 (4.2)	NS
**31–40**	31 (11.6%)	6.4 (4.3)	
**41–50**	68 (25.4%)	5.0 (4.0)	
**51–60**	91 (34.1%)	4.6 (4.2)	
**> 60**	60 (22.5%)	4.9 (3.8)	
**Type of practice**			
**Solo**	120 (44.9%)	4.7 (4.2)	NS
**Duo**	60 (22.5%)	6.1 (4.1)	
**Group**	83 (31.1%)	5.2 (3.9)	
**Missing**	4 (1.5%)		
**Pall care education**			
**Yes**	59 (22.1%)	5.3 (4.1)	NS
**No**	203 (76.0%)	5.2 (4.1)	
**Missing**	5 (1.9%)		
**RIPLS score**			
**Mean**	85.2	B: 0.058	0.024
**Standard deviation**	10.2	95% CI: 0.008; 0.108	
	**PHCT nurses (n = 73)**	**Mean (SD)**	**p**
**Gender**			
**Male**	14 (19.2%)	5.6 (4.2)	NS
**Female**	57 (78.1%)	4.5 (3.6)	
**Missing**	2 (2.7%)		
**Age category**			
**< 31**	9 (12.3%)	5.1 (3.3)	NS
**31–40**	28 (38.4%)	4.6 (3.6)	
**41–60**	32 (43.8%)	4.2 (4.0)	
**> 60**	4 (5.5%)	6.2 (5.0)	
**Years in practice**			
**Mean**	7.15	B: −0.176	0.041
**Standard deviation**	5.1	95% CI: −0.345; −0.00	
**RIPLS score**			
**Mean**	90.8	B: 0.026	NS
**Standard deviation**	8.0	95% CI: −0.104; 0.157	

NS: not significant.

### Multiple linear regression

For both GPs and PHCT nurses, multiple linear regression showed that only gender significantly influenced the number of learned items with male GPs learning less than female GPs and male nurses learning more than female nurses. The effects of years in practice (for the PHCT nurses) and RIPLS score (for the GPs) as shown in the bivariate analysis are not confirmed in the multiple linear regression analysis.

Table 4 shows the results of the multiple linear regression.

**Table 4:** **Explained variance of total count of items learned – multiple linear regression**

**GPs**	
Independent variables	Gender, Age category, Practice organization, Pall. Care education, RIPLS score
Model statistics	Adj R^2^ = 0.07, p = 0.004
**PHCT nurses**	
Independent variables	Gender, Age category, Years in practice, RIPLS score
Model statistics	Adj R^2^ = 0.01, p = 0.386

Independent variables: gender (male/female), age category (<31, 31–40, 41–50, 51–60, >60), RIPLS score (continuous), type of practice (1: solo practice, 2: duo practice, 3: group practice), previous palliative care education (yes/no), years in practice (continuous).

## Discussion

Since there is no well-designed mandatory undergraduate education in palliative care for medical students in Belgium and as the current offer of continuing medical education (CME) in palliative care shows to be insufficient, the exploration of complementary workplace learning merits attention [25]. Our study shows that GPs and PHCT nurses do learn during inter-professional collaboration, thereby confirming that working and learning are inseparable as found in the literature.

In answer to our first research question, both GPs and PHCT nurses learn more about patient related topics (physical and psychosocial) than non-patient related topics (e.g., teamwork, palliative care organization), although all topics are mentioned. This is in line with national surveys on quality of dying and the difficulties in controlling patients’ symptoms in the final stage of life, thereby identifying physicians’ learning needs on this [26]. GPs’ preferences towards palliative care education confirm the importance of dealing with patient related symptoms [27,28]. However, other research identifies care coordination as a major learning need for GPs [29]. Our study participants do not mention teamwork and collaboration as something they learn through collaboration. This needs further attention as the current CME offer is equally insufficient on these two topics [25]. Communication as a means to optimize both teamwork and care coordination is an important learning need in some studies and might inspire communication trainers [30].

The second research question examines the way participants learned through collaboration. The most mentioned learning activities were ‘listening and observing’ and ‘discussion and reflection’. Both activities can be part of the daily collaboration between professionals and are therefore easy-to-use learning activities. Because these activities are part of the normal interactions between professionals, they might not be acknowledged as being learning activities. Our study shows that professionals, without specific training in WPL, are able to recognize these activities as learning strategies, when asked about it. Training professionals to be attentive toward these strategies might optimize the effectiveness of WPL. Other strategies, like ‘receiving feedback’ and ‘learning from mistakes’, are known to be effective educational strategies [31,32]. It is regrettable that they are used less often. However, a high level of trust is required between team members to use practice mistakes as learning moments. A health care team with ever changing members, as often occurs in primary care, should make special efforts to accomplish this since ‘mutual performance monitoring’ is a core component to successful teamwork, leading to continuing improving quality in health care delivery [33,34]. Receiving training in techniques of clinical incident analysis could be useful to stimulate health care professionals to adopt this way of learning and might also enhance team-functioning [35,36].

In answering the third research question (who they learned from), both GPs and PHCT nurses state they learn most from the patient and his family. This sounds logical since palliative care is very much patient-centered and therefore problems and their solutions are patient-focused. Health care professionals but also educators (undergraduate as well as CME) should be aware of the learning aspect of communicating with patients. As our study shows family members to be a major learning source for professionals, we might regard them as part of the care team. Family members have a major caregiving task and as such have to collaborate with professional caregivers [37]. Making professionals and family members aware of the learning effect of collaboration, might stimulate the latter and provide extra satisfaction. A drawback of the predominant focus on patient-related topics might be that the acquired knowledge is tied up with patient details. Making knowledge transferable to other patient situations requires a de-contextualization of the knowledge which is not always easy [38-40]. GPs also learn from PHCT nurses. Being the experts, the nurses have an advisory role and GPs seem to learn from it. This confirms the results from previous focus-group research where GPs describe the collaboration as a teaching/learning interaction [41]. This is supported by literature, describing the newly qualified doctors’ informal learning from nurses. Our study shows that even experienced doctors (though not all of them and not always) acknowledge the nurses’ expertise and declare they learn from them [42].

In answer to the fourth research question, participants associate different types of learning activities and different persons to learn from, with the learning topic at stake. This indicates that participants are able to switch between different learning activities when needed and find expertise among different stakeholders. This makes sense since certain topics are more suited to certain ways of learning than others, e.g., learning to handle a syringe driver by observing a PHCT nurse versus learning about a patient’s fear through discussions with family members. Our study does not show how participants choose these learning activities and learning sources and whether their choice makes WPL effective or not.

The fifth and sixth research questions consider the amount of learning through collaboration. There is no significant difference between the amount of learning by GPs and by PHCT nurses, although the latter are considered to be the experts. Nurses mostly mention learning psychosocial issues from patients and their families. This might account for the high amount of learning they mention since the individual contextual characteristics of palliative patients require continuous attention and learning despite their general expertise in palliative care. Proxy criteria of high expertise, such as years of experience, age and previous education in palliative care, are not associated with the amount of learning. This can be explained in various ways. Positively we could state that even experienced professionals stay eager to learn and to gain new knowledge and expertise through collaboration, on a deeper level. Negatively we might presume that professionals forget what they have learned and need to ‘learn it again’ on the next occasion. A third hypothesis is that the science of palliative care is quickly evolving and requires continuous learning. Our study however does not allow us to draw conclusions on this. The low adjusted R^2^ of 0.07 and 0.01 indicate that personal characteristics are not the major influencing variables explaining the amount of learning. The statistical significant effect of, e.g., gender must therefore be put in this perspective. It might be worthwhile exploring other variables (e.g. team dynamics and inter-professional relationships) for their influence on the amount and quality of workplace learning.

### Strengths and limitations

Strengths: This is the first study to document what, how and from whom learning occurs through inter-professional collaboration in primary palliative care. A retrospective cross-sectional design enabled reporting of actual WPL through collaboration, since no intervention or information was delivered beforehand which might have interfered with the natural way of collaboration.

Limitations: The design and editing of the Learning Content questionnaire might have had an impact on the results and analysis. There is a substantial difference in the number of items for the various sections, e.g., 29 for psychosocial items versus 2 for team items. This difference originates from the content of the EAPC curriculum suggestions and is therefore content-valid. It is unclear however what the probing effect is of the enumeration of a smaller or larger number of items. The predominance of self-reported physical and psychosocial items learnt might reflect a reality or might be induced by the larger number of these items in the questionnaire. The same bias might be suspected for the item ‘other’ only included for the physical aspect and for the variability in the level of specificity (abstraction) in the wording of items for the various categories. The questionnaire however has been tested (with cognitive interviewing) among GPs and nurses and the wording of all items has proven to be perfectly understandable. As these measurement instruments are new, no further details on reliability and validity are available. Self-reported learning has its limitations. It shows us the participants’ perception of the learning at that moment but it does not guarantee effective learning over time. However our study shows that professionals are open to learn through collaborative practice on many topics. This can inform providers of education on the possibilities for this way of learning. Learning through collaboration is strongly linked to the quality of inter-professional and inter-personal dynamics. This aspect has not been captured in our study. Furthermore we limited this study to GPs (who have final responsibility for patient care in primary care and are considered ‘the learner’ in our study) and the PHCT nurses (the ‘expert’). It would be worthwhile investigating WPL for all other team members. Throughout the years, GPs have built good relationships with PHCT nurses and the literature shows us that good relationships are fundamental in workplace learning. We need to be careful however in transferring the results of our study to other settings without such a history of collaboration. As the questionnaires asked about learning on a patient by patient case, anonymity was not possible. This raises the issue of bias from social desirability. However, GPs and nurses were not able to see each other’s responses as this was an online questionnaire, only accessible by the researchers. An introduction letter guaranteed confidentiality. The RIPLS has been validated, taking formal education and training into account. This is the first time the scale has been used in workplace learning. Therefore it is difficult to interpret the meaning of the fact that the RIPLS score has no effect on the amount of learning.

## Conclusion

Conclusion: Both GPs and PHCT nurses state they learn a lot during collaboration in primary palliative care. Different learning activities are used and all caregivers, professional and non-professional (family members), share their expertise.

Practice implications: Identifying the content of WPL might help providers of training and education adapt their curriculum in an anticipatory way. Getting insight into the characteristics of WPL can inform future studies investigating the effectiveness of it. All health care professionals should be aware of this kind of learning and adopt the attitude of sharing expertise during collaboration.

Future research: The effect of workplace learning has to be objectively assessed by means of measuring competence of health care providers and quality of patient care.

## Additional file

Additional file 1:
**Palliative care symptoms and care aspects as content of workplace learning.**
