# Supplementary material for: What, how and from whom do health care professionals learn during collaboration in palliative home care: a cross-sectional study in primary palliative care
Source: BMC Health Serv Res. 2014 Nov 7;14:501. doi: 10.1186/s12913-014-0501-9 (PMC4226882; doi:10.1186/s12913-014-0501-9)
Supplement: Additional file 1: — Palliative care symptoms and care aspects as content of workplace learning. [file 12913_2014_501_MOESM1_ESM.docx]

This questionnaire concerns your recent collaboration with the palliative home care team.

The list shows relevant symptoms and care aspects of palliative care.

- 1. **Physical aspects**

*Answer ‘yes’ if you have learned something about diagnosis or treatment of each symptom.*

1. Pain
2. Sore mouth
3. Anorexia, cachexia
4. Nausea and vomiting
5. Constipation
6. Intestinal obstruction
7. Swallowing problems
8. Breathlessness
9. Cough
10. Hiccups
11. Anxiety
12. Depression
13. Delirium, acute confusional states
14. Weakness, lethargy
15. Sexual problems
16. Incontinence
17. Hypercalcemia
18. Spinal cord compression
19. Superior vena cava obstruction
20. Massive hemorrhage
21. Wound care, wound care materials (including pressure sores)
22. Management of stomas
23. Raised intracranial pressure
24. Restlessness in the last few days of life
25. Indications for use of syringe driver
26. Syringe driver set up
27. Drug use in syringe drivers – stability and miscibility
28. Other: complete

- 1. **Psychosocial aspects**
     1. Family and social background

I have learned :

1. To question and discuss the views of family members towards patient’s disease and treatment
2. To understand the importance of family meetings by participating in such a meeting
3. To recognize the impact of illness on interpersonal relationships in the family
   - 1. Communication

I have learned :

1. To estimate the patient ’s knowledge on his/her prognosis
2. To conduct a bad news conversation in a way the patient accepts
3. To conduct a bad news conversation in a way the family accepts
4. To adequately respond to the fears of the patient
5. To adequately respond to the fears of family members
6. To give information to the patient appropriate to his/her wishes and needs
7. To involve the patient in discussions on treatment and medical policy
   - 1. Psychological responses to life-threatening illness and loss

I have learned :

1. To handle patient s’ grief
2. To handle family members’ grief
3. To discuss ‘hope’ with the patient other than ‘hope to be cured’
4. To handle specific needs of children
5. To handle feelings of anger from patients and family members
6. To handle feelings of guilt with patients and family members
7. To handle feelings of denial with patients and family members
8. To handle ‘conspiracy of silence’

- - 1. Sexuality

I have learned:

1. To handle the changed self-perception of patient towards his body
2. To handle sexual problems with patients and family members
   - 1. Grief

I have learned:

1. To support someone in grief
2. To prepare family members to grief
3. To recognize complex and pathological grief
4. To recognize the special needs of grief with children
   - 1. Being aware of personal and professional feelings

I have learned:

1. To recognize and handle my own emotional stress
2. To recognize and handle emotional stress of team members
3. To respect that others’ values and belief systems can be different than mine
4. To handle my own feelings of guilt after shortcomings in care delivery
5. To recognize and acknowledge the effect of personal loss and grief on (quality of ) care delivery
   1. **Religious, cultural and ethical aspects**

I have learned:

1. To handle patients’ spiritual needs
2. To handle the impact of religious and cultural background towards preferences of care delivery
3. To discuss treatment options with the patient and jointly writing a care plan
4. Not to keep information from the patient on a third person’s request
5. To respect and acknowledge a patient’s wish to refuse treatment
6. The legal aspects of questions towards active life ending
7. To explore a euthanasia request and accompany the patient during his journey

- 1. **Multidisciplinary teamwork**

I have learned:

1. To respect the skills and contributions of other members of the multiprofessional team
2. To share my own tasks and responsibilities with other team members when appropriate
   1. **Organization of care**
      1. Legal frameworks

I have learned:

1. What the procedures for relatives are following a death
2. How cultural aspects can influence procedures after death
3. To know how to access benefits, grants and allowances available to patients and families
   - 1. Practical support for patients and families

I have learned:

1. How other settings (hospital, palliative care unit, home for the elderly) can offer high quality palliative care
2. How to access practical help for the patient (e.g. hospital beds, mattresses)
3. How physiotherapy can benefit the patient
4. How to access supplementary support for the patient (day care, night care, volunteers)
